# Premammalian origin of the sperm‐specific Slo3 channel

**DOI:** 10.1002/2211-5463.12186

**Published:** 2017-02-17

**Authors:** Alberto Vicens, Karla Andrade‐López, Diego Cortez, Rosa María Gutiérrez, Claudia L. Treviño

**Affiliations:** ^1^ Departamento de Genética del Desarrollo y Fisiología MolecularInstituto de BiotecnologíaUniversidad Nacional Autónoma de MéxicoCuernavaca MorelosMéxico; ^2^ Centro de Ciencias GenómicasUniversidad Nacional Autónoma de MéxicoCuernavaca MorelosMéxico; ^3^ Departamento de Microbiología MolecularInstituto de BiotecnologíaUniversidad Nacional Autónoma de MéxicoCuernavaca MorelosMéxico

**Keywords:** evolution, gene loss, ion channels, Slo3, sperm, vertebrates

## Abstract

Slo3 is a sperm‐specific potassium (K^+^) channel essential for male fertility. Slo3 channels have so far been considered to be specific to mammals. Through exploratory genomics, we identified the *Slo3* gene in the genome of terrestrial (birds and reptiles) and aquatic (fish) vertebrates. In the case of fish, *Slo3* has undergone several episodes of gene loss. Transcriptomic analysis showed that vertebrate Slo3 transcript orthologues are predominantly expressed in testis, in concordance with the mammalian Slo3. We conclude that the *Slo3* gene arose during the radiation of early vertebrates, much earlier than previously thought. Our findings add to the growing evidence indicating that the phylogenetic profiles of sperm‐specific channels are intermittent throughout metazoan evolution, which probably reflects the adaptation of sperm to different ionic milieus and fertilization environments.

**Abbreviations**
BK: big potassium
Em: membrane potential
RPKM: reads per kilobase of transcript per million mapped reads
sNHE: sperm‐specific sodium–hydrogen exchanger
TetraCNGK: Tetrameric cyclic nucleotide‐gated potassium channel

Regulation of membrane potential (Em) is vital for sperm function and, consequently, for fertility. Membrane hyperpolarization is an essential physiological change in sperm that enable it to acquire fertilization capacity 1, 2. This signalling event is conserved in phylogenetically distant species such as mammals 3 and marine invertebrates 4. Such hyperpolarization is principally caused by K^+^‐selective channels. Nonetheless, the identity and functional properties of K^+^ channels as well as the molecular targets of sperm membrane hyperpolarization vary greatly among lineages. For instance, in the sea urchin, sperm membrane hyperpolarization is caused by a cyclic nucleotide‐gated K^+^ channel (TetraCNGK) expressed in sperm flagellum 4, 5, triggering a calcium (Ca^2+^) influx via activation of Catsper, a sperm‐specific Ca^2+^ channel that controls sperm chemotaxis 6. TetraCNGK‐mediated hyperpolarization also occurs in the sperm of the freshwater fish *Danio rerio*, but in this species the TetraCNGK channel is located in the sperm head, and its activity is controlled by intracellular pH (pH_i_) rather than by cyclic nucleotides 7. In mammals, the principal K^+^ current that triggers the hyperpolarization of sperm membrane during capacitation, an essential process that prepares sperm to fertilize the ova, is generated by Slo3, a pH_i_‐sensitive K^+^ channel located in the principal piece of sperm flagellum 8, 9, 10, 11, 12, 13. In mouse, it has been demonstrated that the activity of the Slo3 channel is essential for male fertility 10, 11 and that membrane hyperpolarization is necessary and sufficient to prepare sperm for acrosome reaction 14. Slo3 belongs to the family of high‐conductance potassium channels, also known as big potassium (BK) or Slo channels 15. The most studied member of this family is Slo1, a Ca^2+^ and voltage‐activated K^+^ channel essential for excitability of both neuronal and non‐neuronal cells 16. Slo1 is widely distributed across metazoan and is the closest paralogue to Slo3 15. Given that mammals are the sole species that undergo sperm capacitation, it has been widely assumed that Slo3 channels are only found in mammalian sperm membrane. Nonetheless, the phylogenetic distribution of the Slo3 channel has not been rigorously explored to date. Therefore, to better understand the functional significance of the Slo3 channel, in this study, we assess the evolutionary origin and species distribution of the *Slo3* gene using a phylogenomic approach that involves comparative genomics and transcriptomic analyses.

## Materials and methods

### Genomic databases mining

Search of the *Slo3* gene (annotated in genomic databases as *Kcnu1*) in amniotes was performed in the National Center for Biotechnology Information (NCBI) database (http://www.ncbi.nlm.nih.gov/). *Slo3* gene sequences from *Homo sapiens* and *Gallus gallus* were used as initial queries in BLAST searches against genome databases from Ensembl, NCBI and the Joint Genome Institute (http://www.jgi.doe.gov/). Protein sequences were employed for BLASTp and tBLASTn searches against amino acid and nucleotide (i.e. EST, mRNA, genome assembly) databases respectively. Nucleotide‐coding sequences were used for BLASTn and tBLASTx searches as only RNA sequences read archives were available. To identify potential distant homologues, we performed BLAST analysis employing sequences identified during the first round of searches. To identify putative homologues in species where BLAST searches did not yield significant hits and the *Slo3* gene has not been annotated, we scanned the genomic sequence between the two adjacent genes using three *ab initio* gene prediction tools – AUGUSTUS 17, Geneid 18 and FGENESH 19 – and predicted coding sequences were blasted against bony vertebrates database (taxid: 117571). Mapping and comparison of syntenic regions was performed with the Ensembl Compara toolkit and the Genomicus database 20.

### Sequence retrieval, multiple alignment and phylogenetic analyses

Protein sequences were retrieved from NCBI and UniProt (http://www.uniprot.org/) resources and assembled into a multiple sequence alignment. A protein alignment was performed with the Muscle algorithm implemented in geneious R6.1 (Biomatters, http://www.geneious.com/). The alignment was then manually inspected and refined. Protein alignment was used to build a phylogenetic tree. Selection of the best‐fit model of protein evolution was performed with the model selection tool implemented in mega 6.0 21. Phylogeny was generated by the maximum likelihood method using mega, performing a bootstrap test of 100 replications. Slo3 phylogenies of mammalian species, birds and reptilian species were performed by Neighbour‐Joining method implemented in geneious using Slo1 sequences as the outgroup.

### Gene expression data

For amniotes, we downloaded RNAseq fastq files from the SRA database for the selected species (BioProject PRJNA143627). We mapped the RNA‐seq reads with TopHat 2 using the reference genomes of Ensembl release 31 and then used Cufflinks 2 (all mapped reads, embedded multiread and fragment bias correction) to calculate the RPKM (reads per kilobase of transcript per million mapped reads) values for the *Slo3* gene in the available tissues. We normalized expression levels across samples and species with a median scaling procedure. For spotted gar, we retrieved RNAseq data from different tissues (BioProject PRJNA247500) and used the genome assembly LepOcu1 22 as reference genome.

## Results

### Conservation of Slo3 channel in amniotes

**Figure 1:**
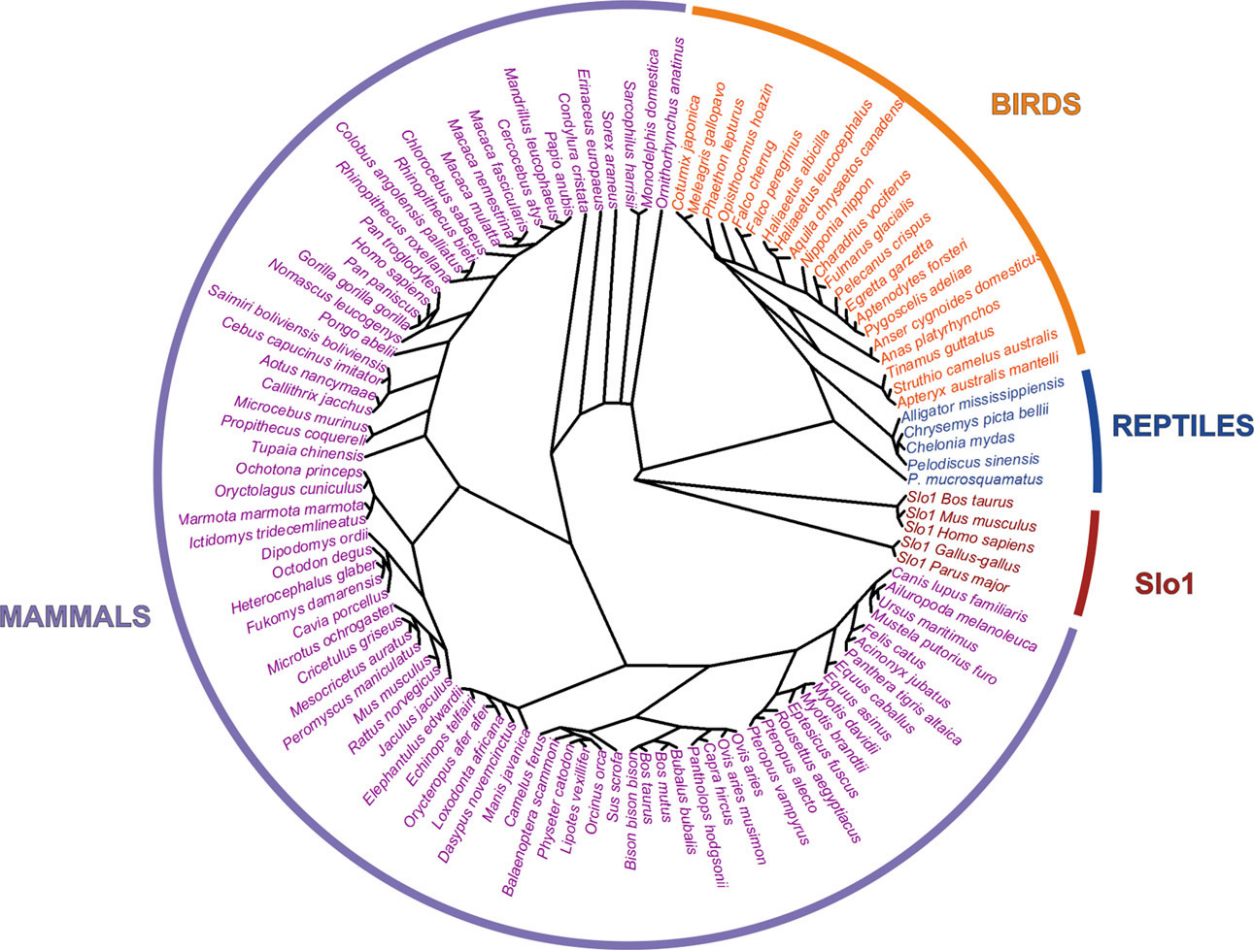
Phylogenetic tree of Slo3 in amniotes. A cladogram built with Slo3 protein sequences from 80 mammalian, 21 birds and 5 reptilian species is represented. Slo1 sequences from human, mouse, bovine, chicken and great tit were used as outgroup.

**Figure 2:**
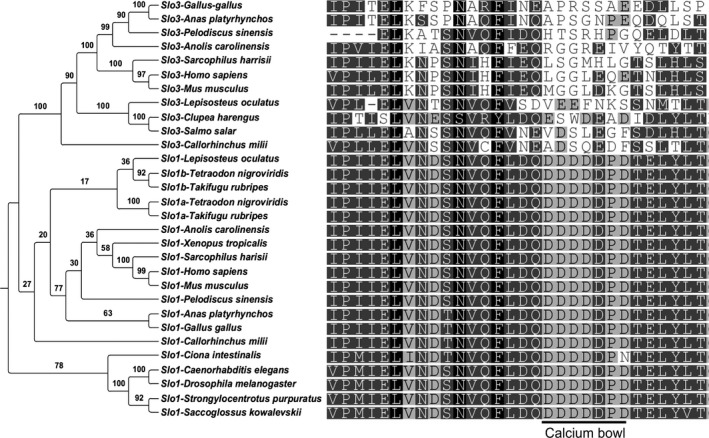
Comparative analysis of Slo1 and Slo3 sequences. Cladogram representing the molecular phylogeny built with Slo1 and Slo3 protein sequences. Phylogenetic tree was built by Maximum Likelihood, and supporting values of posterior bootstrap analysis with 100 replicates are shown on each node. The model of protein evolution used was JTT+G (G fixed to 0.937). Sequence alignment of the calcium bowl region corresponding to Slo1 is shown on right. Amino acids are coloured indicating similarity with < 60% (white), 60–80% (light grey), 80–99% (dark grey) and 100% (black). The residues involved in Ca^2+^ sensing in Slo1 are indicated with a black line under alignment.

**Figure 3:**
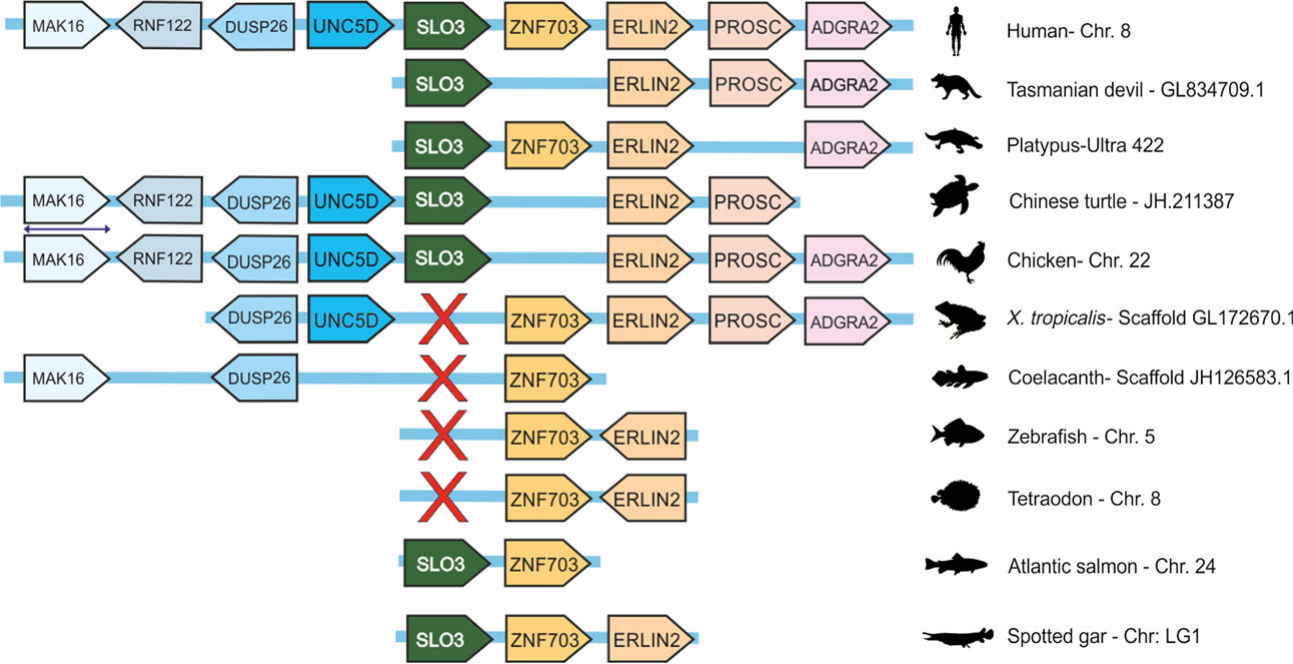
Synteny of *Slo3* locus in vertebrates. The human *Slo3* gene in chromosome 8 was used as reference of syntenic chromosomal regions. *Slo3* gene is represented in the central block and the eight flanking genes (four on either side) as blocks with scaled dark and light colours. Blocks are shown with the direction of gene sequences (right: 5′→3′; left: 3′→5′). Red crosses indicate absence of *Slo3* gene.

We tested the assumption that Slo3 channel is found in mammalian sperm by searching *Slo3* gene in the NCBI database. We found *Slo3* gene orthologues in 80 species spanning the mammalian phylogeny (Fig. 1, Table S1). In a further search of amniote database we found the *Slo3* gene in a wide range of birds and reptiles (Fig. 1, Table S1). We verified the orthology of these sequences applying the following criteria: (a) monophyletic grouping of all sequences within the Slo3 clade in a phylogenetic tree built with Slo1 and Slo3 protein sequences (Figs 1 and 2); (b) the lack of the Ca^2+^‐binding site (calcium bowl) that is uniquely present in the Slo1 channel (Fig. 2); (c) the conservation of synteny of the *Slo3* locus with those of mammalian genomes (Fig. 3). These findings indicate that the *Slo3* gene is present in the genome of all major groups of amniotes.

### Identification of *Slo3* orthologues in aquatic species

In order to determine the evolutionary origin of the *Slo3* gene, we further searched in genomic databases of amphibians and fishes (Table S2). We only found a *Slo1* homologue gene in the genome of the frog *Xenopus tropicalis* (Fig. S1), the amphibian species whose complete genome has been sequenced. We then screened the genomic sequence presumably comprising the *Slo3* locus and interestingly we identified three small intronic sequences highly conserved with human Slo3 (Fig. S1). This suggests that the *Slo3* gene could have developed in Xenopus, but rapidly degenerated. We next examined the transcriptome of the lungfish *Protopterus annectens*, representing the closest lineage relative to amniotes 23. We identified four short reads that map to highly conserved regions between Slo1 and Slo3 with which we cannot determine the presence of *Slo3* gene in lungfish unambiguously. In the genome of the coelacanth *Latimeria chalumnae*, we uniquely identified a *Slo1* orthologue (Fig. 2, Table S1). Similar to that observed in *Xenopus*, we found three short sequences in the syntenic region of the putative *L. chalumnae Slo3* locus highly conserved with human *Slo3* (Fig. S2). Surprisingly, these sequences do not overlap with those found in Xenopus. This observation adds support to the hypothesis that the *Slo3* gene could have been present in aquatic vertebrates. We next explored the genome of several Teleostei (bony fishes). Interestingly, we identified potential *Slo3* orthologues in two teleost species: *Clupea harengus* and *Salmo salar* (Table S1). These sequences were robustly resolved within the Slo3 clade, lack the poly‐aspartic repeat that defines the Slo1 calcium bowl (Fig. 2), and conserve the synteny with tetrapod genomes (Fig. 3). We did not find hints of the *Slo3* gene in the remaining teleost genomes searched. Nonetheless, conserved synteny of the closest flanking genes suggest that *Slo3* was lost in these lineages (Fig. 3). Teleost fishes have undergone at least a round of whole‐genome duplication and several *Slo1* gene copies have been identified (Table S1) 24. Despite several copies of neighbour genes being detected in the examined teleost genomes (Table S3), we found a single *Slo3* copy in *C. harengus* and *S. salar*. This suggests that additional *Slo3* copies originated by genome duplication have degenerated in these species. In the genome of the spotted gar (*Lepisoteus oculatus*), whose lineage diverged from teleost fishes before their genome duplication event, we identified two *Slo*‐like homologues. Although one of them is a conserved *Slo1* orthologue, the other sequence leads a robustly resolved branch within the Slo3 clade and lacks the poly‐aspartic repeat that defines the Slo1 calcium bowl (Fig. 2). In addition, we detected that the synteny of the chromosomal region spanning this *Slo3*‐like gene is conserved with respect to vertebrate genomes (Fig. 3). These observations support the notion that spotted gar genome conserves a *Slo3* orthologue. Since a high‐throughput genomic or transcriptomic data for other gars is not available, we explored the transcriptome of bowfin (*Amia calva*), whose lineage forms a monophyletic clade (Holostei) with gars 22. Similar to lungfish, we found two RNA reads that align to regions conserved between Slo1 and Slo3, whereby we have no evidence to unambiguously identify a *Slo3* orthologue. We further scanned the genome of the elephant shark (*Callorhinchus milli*), whose lineage – cartilaginous fishes – is phylogenetically the oldest of the living jawed vertebrates, and we found two genomic fragments that form a conserved *Slo3* orthologue as they were assembled (Fig. 2, Table S1). We further searched in the genome of a jawless vertebrate, the sea lamprey (*Petromyzon marinus*), and we identified two partial *Slo*‐like sequences after assembling several genomic scaffolds (Table S1). Nevertheless, both sequences align to a conserved region of Slo channels (Fig. S3) and were too short to be robustly resolved in the phylogenetic tree. In addition, scaffolds of sea lamprey genome are still poorly annotated, for which we could not perform a comparative genomics approach to locate the syntenic regions. Nonetheless, the observation that Metazoan generally have a single *Slo1* copy, excepting teleost with duplicated genomes 22, suggests that jawless vertebrates might preserve an ancestral form of *Slo3*. Genomic searches of remaining chordates and deuterostomes – the cephalochordate *Branchiostoma floridae*, the urochordate *Ciona intestinalis*, the echinoderm *Strongylocentrotus purpuratus* and the hemichordate *Saccoglosus kowalevskii* – yielded solely *Slo1* orthologues (Fig. 2, Table S1). Likewise, we found only a *Slo1* copy in the invertebrate genomes of *Caenorhabditis elegans* and *Drosophila melanogaster* (Fig. 2, Table S1). Altogether, these data suggest that the Slo3 channel emerged at the time of the radiation of early vertebrates, which is much earlier than previously assumed.

### *Slo3* is predominantly expressed in testis of vertebrates

**Figure 4:**
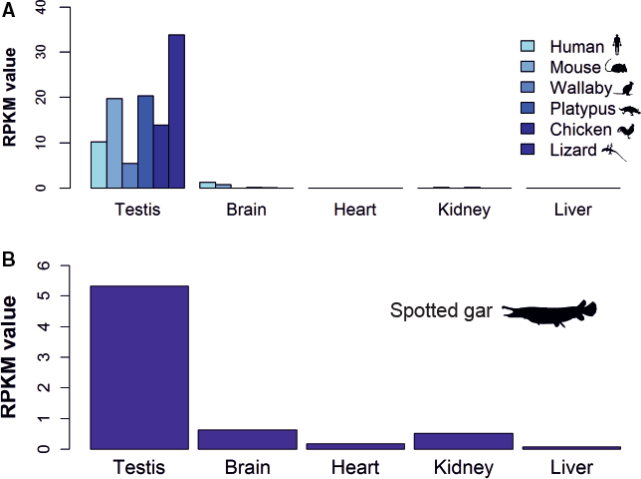
Expression profile of Slo3. (A) Transcript reads of Slo3 from different tissues are shown for a range of representative terrestrial vertebrate species. (B) Expression profile for spotted gar is shown separated as the gene expression data set used for normalization of sequence reads was different to that used for amniotes. Quantitative data are shown as reads per kilobase of transcript per million (RPKM).

To assess whether the expression pattern of Slo3 channel, characterized by a predominant expression in testis 25, is conserved in nonmammalian lineages, we analysed the transcriptome of a range of representative vertebrate species (see Materials and methods). We determined that *Slo3* gene is predominantly transcribed in the testis of model species of birds (chicken) and reptiles (anole lizard) (Fig. 4A). We also identified an enriched expression in the testis of the spotted gar, which was used as the representative fish species (Fig. 4B).

## Discussion

**Figure 5:**
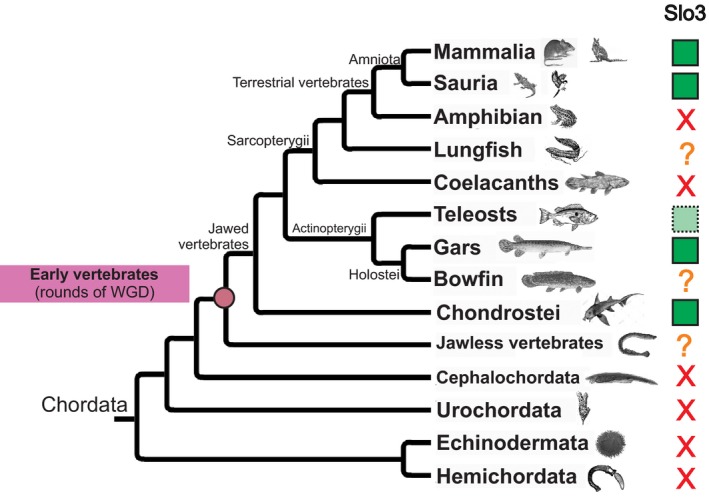
Phylogenetic profile of Slo3 channels. A vertebrate phylogeny is represented describing the presence or absence of *Slo3* gene in examined genomes. Tree topology was based on information from Tree of Life Web Project (http://tolweb.org/tree/). Lineages where *Slo3* gene was identified are indicated by green boxes, while those that lack channels are shown as red crosses. The question marks indicate lineages whose genome information is based on genomic traces or transcriptomic data, and thus the presence/absence of Slo3 cannot be unambiguously interpreted. Light boxes with dashed lines indicate that only some teleost species have Slo3. The ancestral node where vertebrate whole‐genome duplications likely occurred is indicated.

In this study, we found that the emergence of the *Slo3* gene, which encodes a sperm‐specific K^+^ channel, dates to the radiation of ancestral vertebrates (Fig. 5). This finding radically changes the widely established assumption in the field of reproductive biology and sperm physiology that Slo3 is a channel exclusively found in mammals 10, 11, 25. The fact that *Slo3* gene is not only present in the genome of nonmammalian species but also has an active and prominent expression in testis, suggests that the function of Slo3 as a sperm potassium (K^+^) channel is conserved in vertebrates.

We found that the *Slo3* gene is conserved in birds and reptiles. The molecular changes that sperm undergo in the female genital tract are scarcely known in these lineages 26, 27. Our findings place the Slo3 channel as the first described molecular component putatively involved in sperm function of birds and reptiles. Although it is frequently assumed that sperm capacitation is a process confined to mammals, some studies have reported that nonmammalian species undergo sperm capacitation‐like changes after ejaculation 28, 29, 30, 31. These observations, in conjunction with our results, suggest that Slo3 channels could mediate a primary K^+^ current essential to acquire fertilizing ability also in the sperm of nonmammalian vertebrates. Another possibility is that in nonmammalian species, Slo3 channels could have functions in sperm other than mediating membrane hyperpolarization during capacitation‐like processes. For example, Slo3 channels may have a role in thermo‐ or chemosensation responding to sensory cues such as temperature or chemical substances. Functional studies on sperm of avian and reptilian species will be necessary to characterize the role of Slo3 channels in nonmammalian sperm.

An interesting finding of the present work is that the Slo3 channel shows an intermittent pattern of presence and absence across aquatic vertebrates as a result of different events of gene loss. We identified *Slo3* orthologues in some species of bony and cartilaginous fishes, while in a large number of teleost fish species the Slo3 channel appears to have degenerated. In some fishes, orthologues of TetraCNGK, an ancestral and sperm‐specific cyclic nucleotide‐gated K^+^ channel which is also found in marine invertebrates and unicellular eukaryotes 5, 32, have been identified 7. The alternative distribution of Slo3 and TetraCNGK observed across fishes could be the result of lineage‐specific evolutionary conflicts between these two channels for being the principal sperm K^+^ channel. This would explain the presence of TetraCNGK and the absence of Slo3 in coelacanth, or the opposite in the case of elephant shark. Under this hypothetical framework, the conservation in the spotted gar genome of both Slo3 and TetraCNGK 7 is surprising. The coexistence of two sperm‐specific K^+^ channels could still be explained by a subfunctionalization process whereby sperm membrane potential is regulated by the joint activity of both channels. Regarding fishes in which neither Slo3 nor TetraCNGK channels have been identified, it will be worth performing studies encompassing molecular and physiological approaches in order to characterize the K^+^ transporters in these species. The same is true for amphibian species, where the physiological changes that sperm undergo prior to fertilization are poorly understood, and consequently we do not have a clear interpretation about the functional and evolutionary significance of lacking both Slo3 and TetraCNGK channels.

The *Slo3* gene likely originated from a duplication of its closest paralogue *Slo1* at the time of one of the two whole‐genome duplications occurring before divergence of lamprey from the jawed vertebrates (Fig. 5) 33, 34. This seems reasonable as the complex genomic rearrangements that occurred during ancestral vertebrate evolution implied a further expansion of a large number of ion channel families 35, 36. On the other hand, lineage‐specific gene loss seems to be an evolutionary characteristic of sperm‐specific ion channels. Cai and Clapham 37 were the first to observe an heterogeneous phylogenetic profile of the Catsper channel complex in metazoan genomes. In addition, the sperm‐specific sodium–hydrogen exchanger required for sperm motility 38 also shows such a mosaic distribution in metazoans (F. Romero & T. Nishigaki, personal communication). This evolutionary pattern is at odds with that of most ion channels expressed in somatic cells, which are highly conserved across metazoans 36, 39, 40, 41. As a recent review highlights, sperm from different species use distinct types of signalling molecules and highly conserved proteins may perform different tasks according to the fertilization strategy of a particular species 42. Therefore, further evolutionary genomics studies on sperm ion channels will be of great importance to unravel the functional basis of lineage‐specific sperm adaptations throughout metazoan evolution.

## Conclusions

In this study, we established that Slo3, a sperm‐specific channel thought to be mammalian specific, arose with the radiation of early vertebrates. In addition, we observed that the *Slo3* gene has undergone different events of gene loss through evolution of aquatic vertebrates, a feature shared with other sperm‐specific ion channels. The fact that Slo3 channel is widely preserved in terrestrial vertebrates, along with its importance for mammalian fertility, prompt us to suggest that Slo3 channels have a pivotal role in species with internal fertilization. Meanwhile, in aquatic vertebrates that use external fertilization, Slo3 could be evolving under more relaxed functional constraints, resulting in many cases in gene loss. Our findings, together with evidences from recent studies, establish a new picture where channel repertories and functions greatly vary among species, which likely reflects sperm adaptions to species‐specific fertilization environments.

## Author contributions

AV performed genomic database mining, phylogenetic and transcriptomic analysis. KL and RMG initiated the database mining analyses and participated in the drafting of study. DC performed transcriptomic analysis. CT participated in the design, drafting and coordination of the study. All authors read and approved the final manuscript.

## Supporting information


**Fig. S1.** Exploration of *Slo3* locus in Xenopus.

**Fig. S2.** Exploration of *Slo3* locus coelacanth.

**Fig. S3.** Identification of *Slo*‐like sequences in sea lamprey genome.

**Table S1.** Table of protein sequences collected in this study.

**Table S2.** Table with public genomic databases searched during this study.

**Table S3.** Copies of genes flanking *Slo3* locus in teleost fishes.
