# Supplementary figures and images for: Premammalian origin of the sperm‐specific Slo3 channel
Source: FEBS Open Bio. 2017 Feb 17;7(3):382–90. doi: 10.1002/2211-5463.12186 (PMC5337896; doi:10.1002/2211-5463.12186)

**Additional file 3.** Synteny of chromosomal regions harboring the Slo3 locus in human and Xenopus.

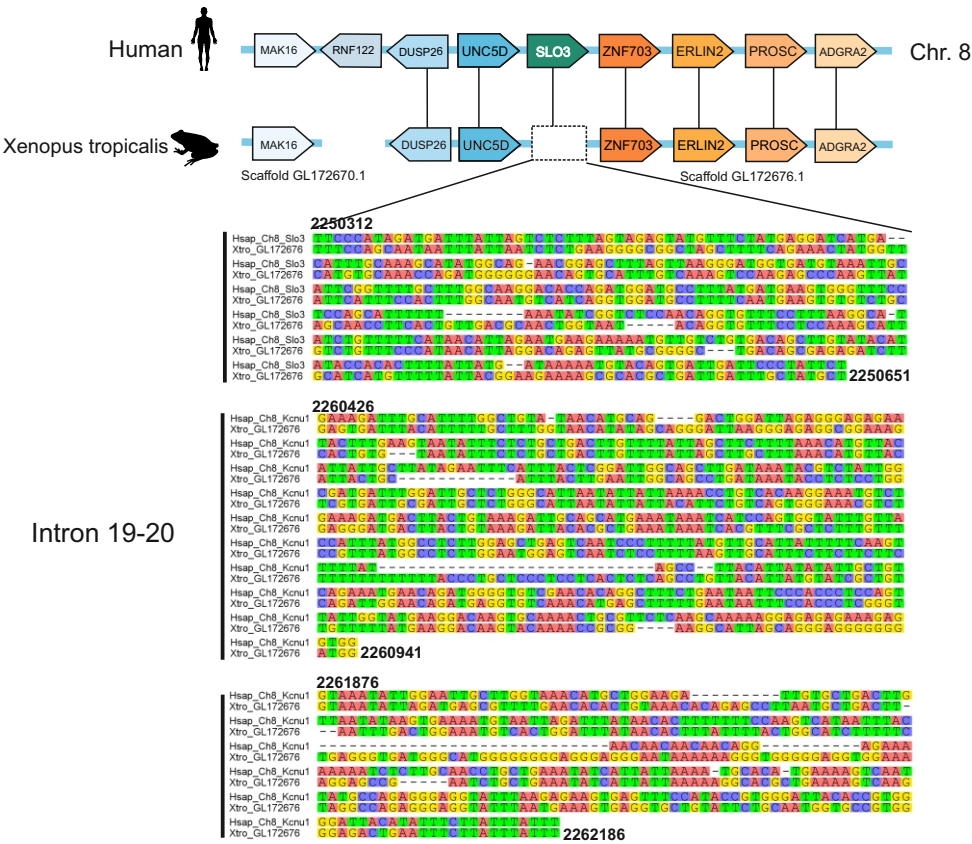

Supplement: Supplementary file 1 — Fig. S1. Exploration of Slo3 locus in Xenopus. [file FEB4-7-382-s001.pdf]
