# Supplementary material for: Premammalian origin of the sperm‐specific Slo3 channel
Source: FEBS Open Bio. 2017 Feb 17;7(3):382–90. doi: 10.1002/2211-5463.12186 (PMC5337896; doi:10.1002/2211-5463.12186)

Additional file 4. Synteny of chromosomal regions haboring the Slo3 locus in human and coelacanth.

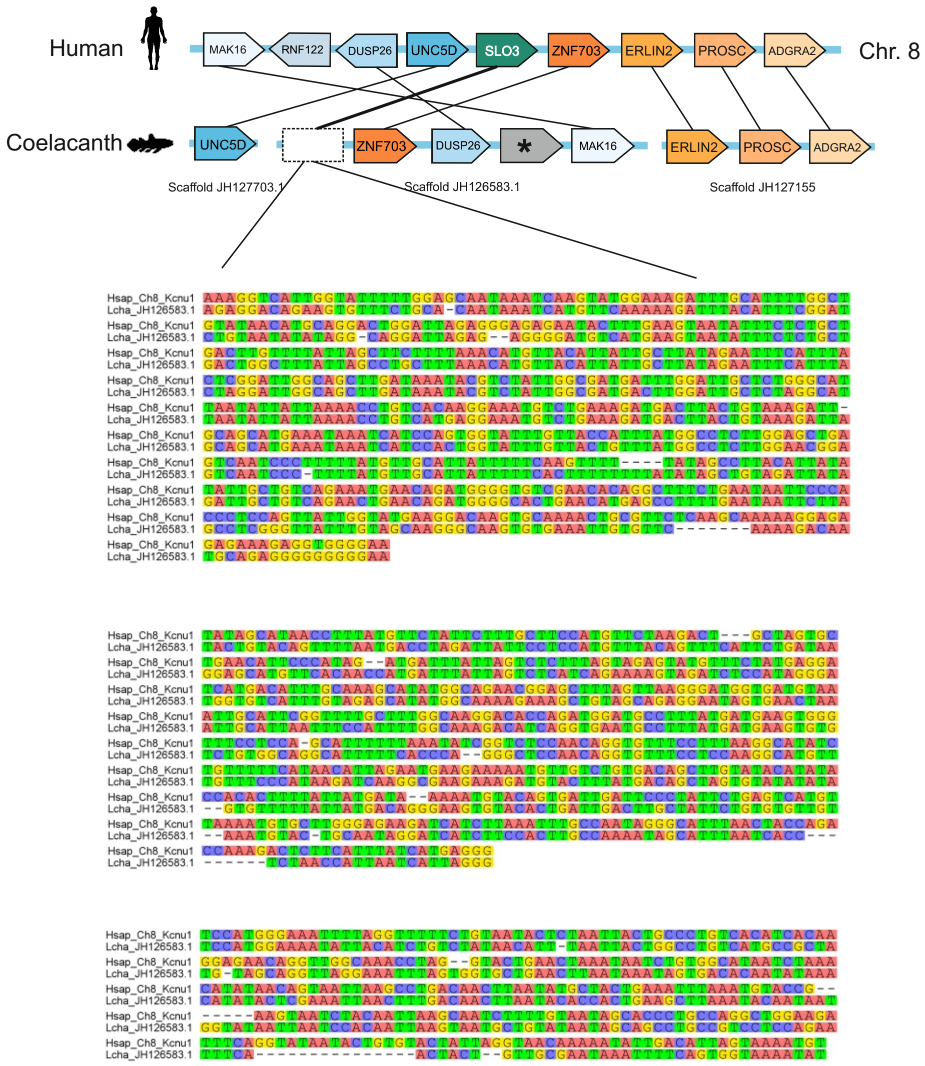

Supplement: Supplementary file 2 — Fig. S2. Exploration of Slo3 locus coelacanth. [file FEB4-7-382-s002.pdf]
