# Supplementary material for: Premammalian origin of the sperm‐specific Slo3 channel
Source: FEBS Open Bio. 2017 Feb 17;7(3):382–90. doi: 10.1002/2211-5463.12186 (PMC5337896; doi:10.1002/2211-5463.12186)

Additional file 6. Identification of Slo-like sequences in the sea lamprey genome.

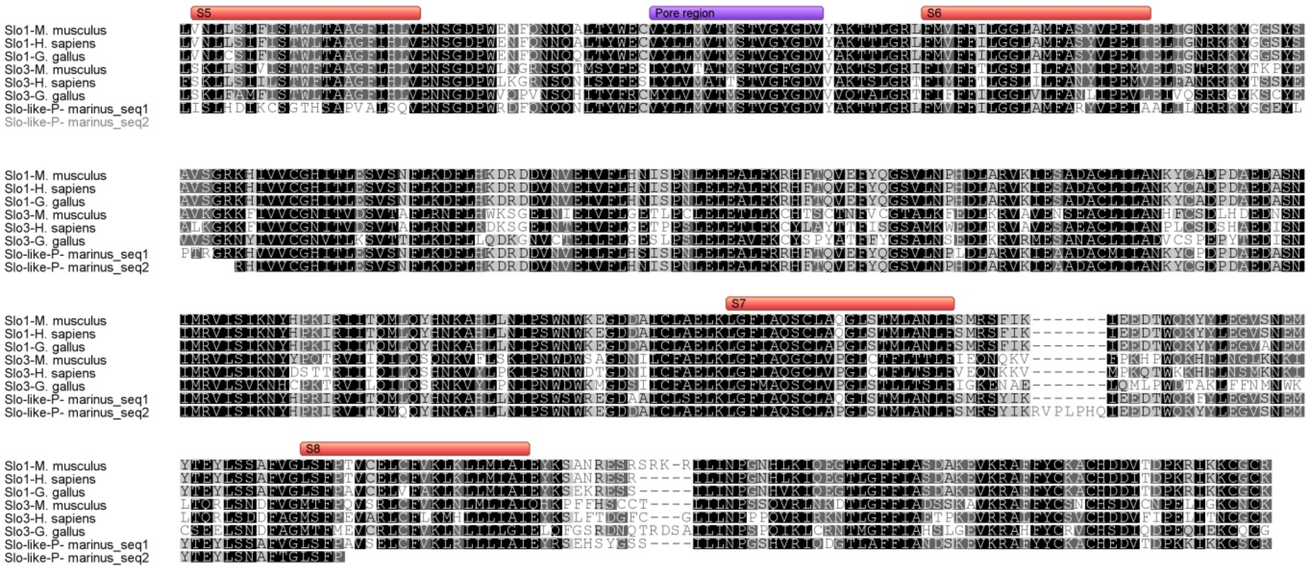

Supplement: Supplementary file 3 — Fig. S3. Identification of Slo‐like sequences in sea lamprey genome. [file FEB4-7-382-s003.pdf]
