# Supplementary material for: Premammalian origin of the sperm‐specific Slo3 channel
Source: FEBS Open Bio. 2017 Feb 17;7(3):382–90. doi: 10.1002/2211-5463.12186 (PMC5337896; doi:10.1002/2211-5463.12186)
Supplement: Supplementary file 4 — Table S1. Table of protein sequences collected in this study. [file FEB4-7-382-s004.pdf]

Additional file 1. List of protein sequences collected in this study

Species	Taxonomic group	Sequence Annotation	Database
**Slo1 sequences**			
Homo sapiens	Mammals	Q12791	Uniprot
Mus musculus	Mammals	Q08460	Uniprot
Sarcophilus harrisii	Mammals	G3WYS3	Uniprot
Ornithorhynchus anatinus	Mammals	F6R266	Uniprot
Gallus gallus	Birds	Q8AYS8	Uniprot
Anas platyrhynchos	Birds	U3J0P9	Uniprot
Anolis carolinensis	Reptiles	H9GDX8	Uniprot
Pelodiscus sinensis	Reptiles	K7F216	Uniprot
Xenopus tropicalis	Amphibian	F6W2H9	Uniprot
Latimeria chalumnae	Lobe-finned fishes	XP_014351282	NCBI GenBank
Clupea harengus	Fishes (Teleostei)	XP_012697367	NCBI GenBank
		XP_012693773	NCBI GenBank
Danio rerio	Fishes (Teleostei)	XP_009305523	NCBI GenBank
		XP_017214139	NCBI GenBank
Esox lucius	Fishes (Teleostei)	XP_010867843	NCBI GenBank
		XP_012989670	NCBI GenBank
Gasterosteus aculeatus	Fishes (Teleostei)	ENSGACP00000002924	Ensemb
		ENSGACP00000011723	Ensemb
Oreochromis niloticus	Fishes (Teleostei)	I3KQC6	Uniprot
		I3KGU3	Uniprot
Oryzias latipes	Fishes (Teleostei)	H2LY41	Uniprot
		H2LYK4	Uniprot
Salmo salar	Fishes (Teleostei)	XP_014067666	NCBI GenBank
		XP_014070975	NCBI GenBank
		XP_014010555	NCBI GenBank
		XP_014035818	NCBI GenBank
Tetraodon nigroviridis	Fishes (Teleostei)	H3D6K3	Uniprot
		HEC4T4	Uniprot
Takifugu rubripes	Fishes (Teleostei)	H2UG69	Uniprot
		H2S8K3	Uniprot
Lepisosteus oculatus	Fishes (Lepisosteiformes)	W5MBE8	Uniprot
Callorhinchus milii	Fishes (Chondrichthyes)	XP_007896678	NCBI GenBank
Ciona intestinalis	Urochordata	F6QI54	Uniprot
Branchiostoma floridae	Cephalochordata	XP_002596143	NCBI GenBank
Strongylocentrotus purpuratus	Echinodermata	W4ZIQ3	Uniprot
Saccoglossus kowalevskii	Hemichordata	XP_006821707	NCBI GenBank
Caenorhabditis elegans	Nematoda	Q95V25	Uniprot
Drosophila melanogaster	Arthropoda	Q03720	Uniprot
**Slo3 sequences**			
Loxodonta africana	Mammals	XP_010590860	NCBI GenBank
Marmota marmota marmota	Mammals	XP_015356705	NCBI GenBank
Ochotona princeps	Mammals	XP_012786287	NCBI GenBank
Panthera tigris altaica	Mammals	XP_007097151	NCBI GenBank
Equus asinus	Mammals	XP_014706863	NCBI GenBank
Balaenoptera acutorostrata scammoı	Mammals	XP_007170167	NCBI GenBank
Eptesicus fuscus	Mammals	XP_008144467	NCBI GenBank
Bison bison bison	Mammals	XP_010837093	NCBI GenBank
Pteropus alecto	Mammals	XP_015448294	NCBI GenBank
Rhinopithecus bieti	Mammals	XP_017704883	NCBI GenBank
Saimiri boliviensis boliviensis	Mammals	XP_010347041	NCBI GenBank
Myotis brandtii	Mammals	XP_005856564	NCBI GenBank
Elephantulus edwardii	Mammals	XP_006896713	NCBI GenBank
Bos taurus	Mammals	NP_001156721	NCBI GenBank
Cebus capucinus imitator	Mammals	XP_017390688	NCBI GenBank
Acinonyx jubatus	Mammals	XP_014931577	NCBI GenBank
Pan troglodytes	Mammals	XP_009453461	NCBI GenBank
Cricetulus griseus	Mammals	XP_003498290	NCBI GenBank
Tupaia chinensis	Mammals	XP_006171561	NCBI GenBank
Pantholops hodgsonii	Mammals	XP_005977316	NCBI GenBank
Colobus angolensis palliatus	Mammals	XP_011816110	NCBI GenBank
Propithecus coquereli	Mammals	XP_012506545	NCBI GenBank
Macaca fascicularis	Mammals	NP_001270492	NCBI GenBank
Fukomys damarensis	Mammals	XP_010602926	NCBI GenBank
Octodon degus	Mammals	XP_004637349	NCBI GenBank
Canis lupus familiaris	Mammals	XP_013975467	NCBI GenBank
Felis catus	Mammals	XP_006944428	NCBI GenBank
Mustela putorius furo	Mammals	XP_012914699	NCBI GenBank
Cavia porcellus	Mammals	XP_013000131	NCBI GenBank
Mandrillus leucophaeus	Mammals	XP_011848411	NCBI GenBank
Rousettus aegyptiacus	Mammals	XP_016010716	NCBI GenBank
Sorex araneus	Mammals	XP_004606998	NCBI GenBank
Ailuropoda melanoleuca	Mammals	XP_002917281	NCBI GenBank
Capra hircus	Mammals	XP_005698879	NCBI GenBank
Mesocricetus auratus	Mammals	XP_012966315	NCBI GenBank
Rhinopithecus roxellana	Mammals	XP_010368920	NCBI GenBank
Microcebus murinus	Mammals	XP_012618385	NCBI GenBank
Monodelphis domestica	Mammals	XP_007476394	NCBI GenBank
Chlorocebus sabaeus	Mammals	XP_007960357	NCBI GenBank
Equus caballus	Mammals	XP_001915699	NCBI GenBank
Mus musculus	Mammals	NP_032458	NCBI GenBank

Homo sapiens	Mammals	NP_001027006	NCBI GenBank
Orcinus orca	Mammals	XP_012394127	NCBI GenBank
Pteropus vampyrus	Mammals	XP_011371619	NCBI GenBank
Jaculus jaculus	Mammals	XP_004671551	NCBI GenBank
Aotus nancymaae	Mammals	XP_012331366	NCBI GenBank
Manis javanica	Mammals	XP_017497404	NCBI GenBank
Ovis aries musimon	Mammals	XP_014961145	NCBI GenBank
Myotis davidii	Mammals	XP_006765714	NCBI GenBank
Heterocephalus glaber	Mammals	XP_004855513	NCBI GenBank
Dasypus novemcinctus	Mammals	XP_012384812	NCBI GenBank
Nomascus leucogenys	Mammals	XP_003269618	NCBI GenBank
Rattus norvegicus	Mammals	XP_006222332	NCBI GenBank
Papio anubis	Mammals	XP_009211137	NCBI GenBank
Dipodomys ordii	Mammals	XP_012879665	NCBI GenBank
Orycteropus afer afer	Mammals	XP_007933245	NCBI GenBank
Sus scrofa	Mammals	XP_013839540	NCBI GenBank
Macaca nemestrina	Mammals	XP_011730984	NCBI GenBank
Ornithorhynchus anatinus	Mammals	XP_007668050	NCBI GenBank
Ursus maritimus	Mammals	XP_008684847	NCBI GenBank
Peromyscus maniculatus bairdii	Mammals	XP_015856995	NCBI GenBank
Microtus ochrogaster	Mammals	XP_005362685	NCBI GenBank
Pan paniscus	Mammals	XP_003830790	NCBI GenBank
Oryctolagus cuniculus	Mammals	XP_017194437	NCBI GenBank
Macaca mulatta	Mammals	XP_015000644	NCBI GenBank
Ovis aries	Mammals	XP_014960085	NCBI GenBank
Echinops telfairi	Mammals	XP_012864190	NCBI GenBank
Cercocebus atys	Mammals	XP_011926040	NCBI GenBank
Physeter catodon	Mammals	XP_007104131	NCBI GenBank
Condylura cristata	Mammals	XP_012580231	NCBI GenBank
Pongo abelii	Mammals	XP_002819037	NCBI GenBank
Sarcophilus harrisii	Mammals	XP_012395415	NCBI GenBank
Ictidomys tridecemlineatus	Mammals	XP_005338461	NCBI GenBank
Bubalus bubalis	Mammals	XP_006042524	NCBI GenBank
Erinaceus europaeus	Mammals	XP_007516791	NCBI GenBank
Gorilla gorilla gorilla	Mammals	XP_018887548	NCBI GenBank
Callithrix jacchus	Mammals	XP_017819085	NCBI GenBank
Camelus ferus	Mammals	XP_014413574	NCBI GenBank
Bos mutus	Mammals	XP_005906171	NCBI GenBank
Lipotes vexillifer	Mammals	XP_007448382	NCBI GenBank
Anas platyrhynchos	Birds	U3JB1	Uniprot
Anser cygnoides domesticus	Birds	XP_013049885	NCBI GenBank
Aptenodytes forsteri	Birds	XP_009284474	NCBI GenBank
Apteryx australis mantelli	Birds	XP_013815616	NCBI GenBank
Aquila chrysaetos canadensis	Birds	XP_011573722	NCBI GenBank
Calidris pugnax	Birds	XP_014813886	NCBI GenBank
Charadrius vociferus	Birds	XP_009882667	NCBI GenBank
Coturnix japonica	Birds	XP_015738668	NCBI GenBank
Egretta garzetta	Birds	XP_009638835	NCBI GenBank
Falco cherrug	Birds	XP_014134568	NCBI GenBank
Falco peregrinus	Birds	XP_013151525	NCBI GenBank
Fulmarus glacialis	Birds	XP_009581603	NCBI GenBank
Gallus gallus	Birds	E1BRT2	Uniprot
Haliaeetus albicilla	Birds	XP_009921563	NCBI GenBank
Haliaeetus leucocephalus	Birds	XP_010561282	NCBI GenBank
Meleagris gallopavo	Birds	XP_010721464	NCBI GenBank
Nipponia nippon	Birds	XP_009467145	NCBI GenBank
Opisthocomus hoazin	Birds	XP_009935858	NCBI GenBank
Pelecanus crispus	Birds	XP_009486747	NCBI GenBank
Phaethon lepturus	Birds	XP_010289621	NCBI GenBank
Pygoscelis adeliae	Birds	XP_009316906	NCBI GenBank
Struthio camelus australis	Birds	XP_009675994	NCBI GenBank
Tinamus guttatus	Birds	XP_010220373	NCBI GenBank
Anolis carolinensis	Reptiles	H9GMG7	Uniprot
Pelodiscus sinensis	Reptiles	XP_006132699	NCBI GenBank
Chrysemys picta bellii	Reptiles	XP_008170424	NCBI GenBank
Chelonia mydas	Reptiles	XP_007053546	NCBI GenBank
Alligator mississippiensis	Reptiles	XP_014458914	NCBI GenBank
Protobothrops mucrosquamatus	Reptiles	XP_015675242	NCBI GenBank
Clupea harengus	Fish (Teleostei)	XP_012671269	NCBI GenBank
Esox lucius	Fish (Teleostei)	NC_025980[a]	NCBI GenBank
Salmo salar	Fish (Teleostei)	XP_014026433	NCBI GenBank
Lepisosteus oculatus	Fish (Actinopterygii)	W5NE14	Uniprot
Callorhincus milii	Fish (Chondrichthyes)	XP_007882802, XP_007882895[b]	NCBI GenBank
Uncharacterized sequences			
Protopterus annectens	Lobe-finned fish	SRR2028017[c]	NCBI SRA
Amia calva	Fish (Actinopterygii)	SRR1524270[c]	NCBI SRA
Petromyzon marinus	Jawless vertebrate	GL_477091 - GL_489054[a,b]	NCBI Assembly
Petromyzon marinus	Jawless vertebrate	GL_486096[a]	NCBI Assembly

[a] Partial coding sequences

[b] Secuences assembled from different scaffolds

[c] Run reference from a Sequence Read Archive (SRA) dataset
