# Supplementary material for: Premammalian origin of the sperm‐specific Slo3 channel
Source: FEBS Open Bio. 2017 Feb 17;7(3):382–90. doi: 10.1002/2211-5463.12186 (PMC5337896; doi:10.1002/2211-5463.12186)
Supplement: Supplementary file 5 — Table S2. Table with public genomic databases searched during this study. [file FEB4-7-382-s005.pdf]

Additional file 2.Table with public genomic databases searched during this study

Species	Common name	Group	Genome reference
Xenopus tropicalis	Western Clawed frog	Amphibian	Xtro JGI 4.2
Protopterus annectens	Lungfish	Fish (Sarcopterygii)	PRJNA282925*
Latimeria chalumnae	West Indian Ocean coelacanth	Fish (Sarcopterygii)	Latcha1
Danio rerio	Zebrafish	Fish (Teleostei)	GRCz10
Takifugu rubripes	Fugu	Fish (Teleostei)	FUGU 4.0
Astyanax mexicanus	Cave fish	Fish (Teleostei)	AstMex102
Oryzias latipes	Japanese rice fish	Fish (Teleostei)	HdrR
Xiphophorus maculatus	Southern platyfish	Fish (Teleostei)	Xipmac4.4.2
Tetraodon nigroviridis	Pufferfish	Fish (Teleostei)	TETRAODON 8.0
Oreochromis niloticus	Nile Tilapia	Fish (Teleostei)	Orenil 1.0
Gasterosteus aculeatus	Three-spined stickleback	Fish (Teleostei)	BROAD S1
Salmo salar	Atlantic salmon	Fish (Teleostei)	ICSASG_v2
Lepisosteus oculatus	Spotted gar	Fish (Holostei)	LepOcu1
Amia calva	Bowfin	Fish (Holostei)	PRJNA255850*
Leucoraja erinacea	Little skate	Fish (Chondrychthyes)	LER_WGS_1
Callorhinchus milii	Elephant shark	Fish (Chondrychthyes)	Sanger AAVX01000000
Petromyzon marinus	Sea lamprey	Hyperoartia	Pmarinus 7.0
Branchiostoma floridae	Florida lancelet, Amphioxus	Cephalochordata	Bflo_1.0
Ciona intestinalis	Vasa tunicate	Urochordata	GCA_000224145
Strongylocentrotus purpuratus	Purple sea urchin	Echinodermata	Spur_3.1
Saccoglosus kowalevskii	Acorn worm	Hemichordata	Skow_1.0
Caenorhabditis elegans	C. elegans	Nematoda	WBcel235
Drosophila melanogaster	Fruit fly	Arthropoda	BDGP5

*BioProjects based on transcriptomic data
