# Supplementary material for: Premammalian origin of the sperm‐specific Slo3 channel
Source: FEBS Open Bio. 2017 Feb 17;7(3):382–90. doi: 10.1002/2211-5463.12186 (PMC5337896; doi:10.1002/2211-5463.12186)
Supplement: Supplementary file 6 — Table S3. Copies of genes flanking Slo3 locus in teleost fishes. [file FEB4-7-382-s006.pdf]

Additional file 5. Copies of genes flanking *Slo3* locus in teleost fishes.

**Species**	**Gene**	**Chromosome**
Danio rerio	Znf703a	Chr 21
	Znf703b	Chr 5
	Unc5da	Chr 21
	Unc5db	Chr 5
Oryzia latipes	Znf703a	Chr 12
	Znf703b	Chr 9
	Unc5da	Chr 12
	Unc5db	Chr 9
Tetraodon nigroviridis	Znf703a	Chr 4
	Znf703b	Chr 12
	Unc5da	Chr 4
	Unc5db	Chr 17
Takifugu rubripes	Znf703a	Scaffold 37
	Znf703b	Scaffold 7
	Unc5da	Scaffold 322
	Unc5db	Scaffold 114
Salmo salar	Znf703a	Chr 24
	Znf703b	Chr 20
	Znf703c	Chr 1
	Znf703d	Chr 11
	Unc5da	Chr 24
	Unc5db	Chr 20
	Unc5dc	Chr 1
	Unc5dd	Chr 11
